# EIF4E regulates STEAP1 expression in peritoneal metastasis

**DOI:** 10.7150/jca.29105

**Published:** 2020-01-01

**Authors:** Jun-nan Jiang, Yuan-yu Wu, Xue-dong Fang, Fu-jian Ji

**Affiliations:** Department of Gastrointestinal Colorectal and Anal Surgery, The China-Japan Union Hospital of Jilin University, Changchun 130033, China.

**Keywords:** STEAP1, gastric cancer, peritoneal metastases, eIF4E.

## Abstract

Gastric cancer is the most prominent form of malignancy in China, and the high mortality associated with it is mostly due to peritoneal metastasis. We have previously elucidated that the RNA-binding protein poly r(C) binding protein 1 (PCBP1) and miR-3978 function as repressors of peritoneal metastasis, partially by downregulation of six-transmembrane epithelial antigen of the prostate 1 (STEAP1). We now show that *STEAP1* is regulated at the level of cap-dependent translation initiation by phosphorylated eIF4E. Chemically inhibiting phosphorylation of eIF4E or genetic ablation of phosphorylated eIF4E inhibit translational upregulation of STEAP1 in the peritoneal metastasis mimicking cell line MKN45 in comparison to the normal mesothelial cell line HMrSV5. Thus phosphorylation of eIF4E is required for peritoneal metastasis of gastric cancer via translational control of *STEAP1*. Chemical inhibitors targeting phosphorylation of eIF4E or its interaction with the translation initiation complex thus might prove effective in treating patients with peritoneal metastasis.

## Introduction

Peritoneal metastasis is the prime cause of mortality associated with gastric cancer, which has an estimated annual incidence of 300,000 patients 1, 2. Even though radical surgical resection has been the management modality it has largely failed to inhibit progression to peritoneal metastasis or decrease the resulting mortality 3-6. Hence, discovering improved biomarkers will potentially aid in both diagnosis and prediction of prognosis of gastric cancer 7, 8.

We have earlier shown that the lysosomal cysteine endopeptidase, legumain - also known as asparaginyl endopeptidase (AEP) - is expressed at higher levels in gastric cancer patients who have peritoneal metastases 9. In fact, legumain has been shown to be overexpressed in a wide variety of tumor types 10-13. Legumain expression in these patients is regulated post-transcriptionally by the microRNA (miR)-3978, which is downregulated in metastatic patients 9. In addition, the tumor suppressor RNA binding protein, poly r(C) binding protein 1 (PCBP1), regulates miR-3978 expression in normal peritoneum and is itself downregulated during metastatic progression, in turn switching on events ultimately resulting in overexpression of legumain 9, 14. Since legumain can potentiate metastatic progression by proteolytic activation of other zymogens or by promoting the epithelial to mesenchymal transition (EMT) via Akt and MAPK signaling 15-18, and suppression of PCBP1 expression or post-translational modification via Akt2-mediated phosphorylation promotes EMT and metastasis via upregulation of specific regulatory proteins and long non-coding RNAs in lung, breast, and gastric cancer 19-23, we enquired the translational landscape of EMT inducers in metastatic gastric cancer and discovered that *STEAP1* (encoding six-transmembrane epithelial antigen of the prostate 1) is translationally upregulated 24. Expression of STEAP1 was required for both tumorigenesis *per se*, as well as for induction of chemoresistance 24.

The goal of the present study was to understand how of *STEAP1* expression in gastric cancer patients is regulated. Who have peritoneal metastases and to define the underlying mechanism(s) of such regulation. We found that *STEAP1* is exclusively regulated at the level of translation initiation of *STEAP1* messenger RNA (mRNA) by phosphorylated eukaryotic initiation factor 4E (eIF4E).

## Materials and Methods

### Patient sample

The Institutional Review Board of the China-Japan Union Hospital of Jilin University approved all aspects of this study protocol. Patients were only enrolled in the current study after providing signed informed consent. From 2014 through 2015, 20 patients (12 men, 8 women) undergoing surgical treatment of gastric cancer in the China-Japan Union Hospital of Jilin University were recruited to the present study. Patients were on average 61.34 years of age (39-78 years). Study inclusion criteria included: peritoneal metastases at the time of diagnosis, no surgical resection, no chemotherapy or radiation therapy, and absence of co-morbidities. Any patient not conforming to one or more of the inclusion criteria were excluded from the current study, Tumor and adjacent normal tissue samples were collected from the gastric tissue of all patients during surgical resection.

### Cell culture and treatment

HMrSV5 and MKN45 cell lines were obtained from the BeNa Culture Collection (Beijing, China). RPMI1640 (Life Technology) containing 20% FBS (Lonza, Germany) was used for all cell culture in a 37^0^C 5% CO_2_ incubator. In the indicated experiments, 10 µM of MG-132 (Sigma-Aldrich, China) was used to treat cells for 8 hours, or 10 µM of CGP57380 (Selleckchem, Houston, TX, USA) was used to treat cells for 24 hours.

### Transfection and transduction

Transfection was performed using Lipofectamine 3000 (Life Technologies, Shanghai, China). ShRNA targeting the 3'UTR of *EIF4E* was obtained from Dharmacon in *pGIPZ* backbone. Lentiviral particles were generated using 293T cells and the Mirus TransIT-293T system (Mirus Bio LLC, USA), based on manufacturer's guidelines. Transductants were selected with 2 µg/mL Puromycin. The wild-type *EIF4E* coding sequence was cloned into pcDNA3.1 and the S209A mutant was generated using site-directed mutagenesis. Once stable knockdowns of *EIF4E* were generated and confirmed, they were transfected with wild-type or S209A mutant *EIF4E* expression plasmid and selected to generate stable clones. Silencing or ectopic overexpression were verified by immunoblotting.

### Western blotting

For cell lysis, lysis buffer containing 25 mM Tris-HCl pH 7.4, 150 mM NaCl, 1 mM EDTA, 1% NP-40, 5% glycerol supplemented with a protease inhibitor cocktail (Roche Diagnostics, Beijing, China) was used. Total protein was separated via SDS-PAGE and blots were probed using anti-STEAP1 antibody (ab3679; Abcam, Waltham, MA, USA), anti-eIF4E antibody (9742, Cell Signaling Technology, Cambridge, MA, USA), anti-P-eIF4E antibody (9741, Cell Signaling Technology, Cambridge, MA, USA). Blots were also probed for β-actin, GAPDH, or HSP90 as indicated to confirm equal loading.

### Quantitative real time polymerase chain reaction (qRT-PCR)

Trizol was used for RNA isolation from tissue specimens and cells. *STEAP1*, *SNAI1*, *MMP9*, *GAPDH*, and *ACTB* expression were detected via TaqMan miRNA assay (Life Technologies), with data being and miRNA data.

### Polysome profiling

Following 30-minute treatment with 100 µg/mL cycloheximide (Sigma-Aldrich) at 37^o^C, cells were washed in cold PBS containing cycloheximide. A buffer containing: 10 mM Tris-Cl, pH 7.4, 5 mM MgCl_2_, 100 mM KCl, 1% (v/v) Triton X-100, 0.5% (w/v) deoxycholate, 1000 U/ml RNasin, 2mM DTT and 100 µg/ml Cycloheximide was used to lyse cells. Lysates were clarified via high speed centrifugation, and then added atop a 10-50% sucrose gradients followed by 100,000g ultracentrifugation for 4 hours in a SW41 rotor (Beckman, USA). Gradient fractionation was performed via BR-184 tube piercer (Brandel, USA) with a UA-6 UV detector (Teledyne ISCO, USA). Data were acquired via DI-158U USB (DATAQ Instruments, USA) and processed based on 254 nm absorption over time using the Peak Chart Data Acquisition Software.

### RNA isolation from polysomal fractions

TRIzol LS reagent (Life Technology) was employed for polysome RNA isolation in accordance with the manufacturer's instructions. RNA was used for qRT-PCR as above.

### Luciferase reporter constructs and luciferase assay

The 3' UTRs were amplified from genomic DNA obtained from HMrSV5 cells. Reporters were sub cloned into the XbaI and ApaI sites of the Renilla Luciferase vector (pRL-CMV CXCR4 6x). The pFR-EMCV (CMV driven firefly and IRES driven Renilla and 3' UTR) were used to generate the bicistronic IRES plasmids. The Dual-luciferase reporter assay system (Promega) was used for all luciferase assays following the manufacturer's protocol on a Tecan M200 multimode reader using Tecan Magellan software (Tecan).

## Results

**Figure 1:**
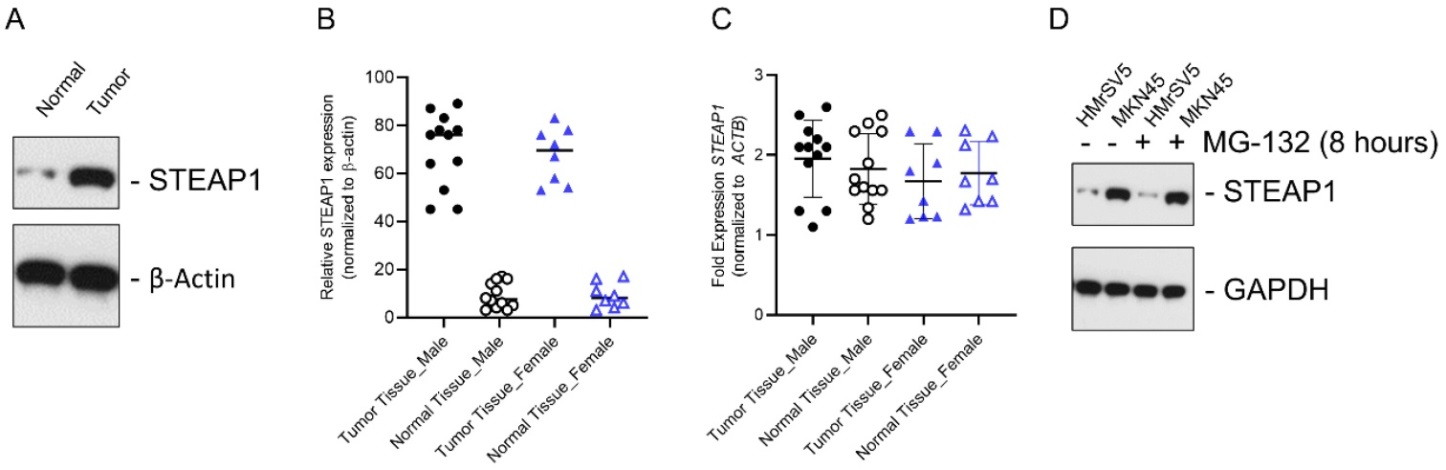
** In gastric cancer patients with peritoneal metastases, *STEAP1* is regulated at the post-transcriptional level. (A)** STEAP1 immunoblot analysis in tumor and normal control samples obtained from gastric cancer patients with peritoneal metastasis. β-actin was used as a loading control. Shown are representative blots. **(B)** Quantification of STEAP1 protein expression shown in **A** in male and female patients. **(C)** Relative *STEAP1* mRNA expression in normal and tumor tissue determined by qRT-PCR, normalizing results to *ACTB* expression. Data points represent all female and male patients included in the current study and are represented as mean ± standard deviation, each sample was done in triplicates. **(D)** STEAP1 immunoblot analysis in the normal mesothelial cell line HMrSV5 and peritoneal metastasis cell line MKN45 ± MG-132 treatment. GAPDH was used a loading control. Shown are representative blots.

We initially determined STEAP1 protein expression in metastatic gastric cancer tissue samples and normal adjacent controls using immunoblot analysis. STEAP1 was significantly overexpressed in tumor tissue (Fig. 1A). The overexpression of STEAP1 was conserved between male and female patients; however, was not significantly different between male and female patients (Fig. 1B; P<0.05 compared to tumor adjacent normal controls). This induction in protein expression was independent of changes in steady state expression of *STEAP1* mRNA in both male and female patients (Fig. 1C), validating our previous finding that *STEAP1* is translationally upregulated in metastatic gastric cancer 24. In order to determine if STEAP1 protein is being made but actively degraded by post-translational regulatory mechanisms, we treated the normal mesothelial cell line HMrSV5 and the peritoneal metastasis mimicking cell line MKN45 with MG-132, which is a proteasomal inhibitor. MG-132 treatment did not result in accumulation of STEAP1 protein in the HMrSV5 or MKN45 cells (Fig. 1C), indicating that post translational degradation mechanism is not responsible for the low STEAP1expression in normal gastric tissue or HMrSV5 cells.

**Figure 2:**
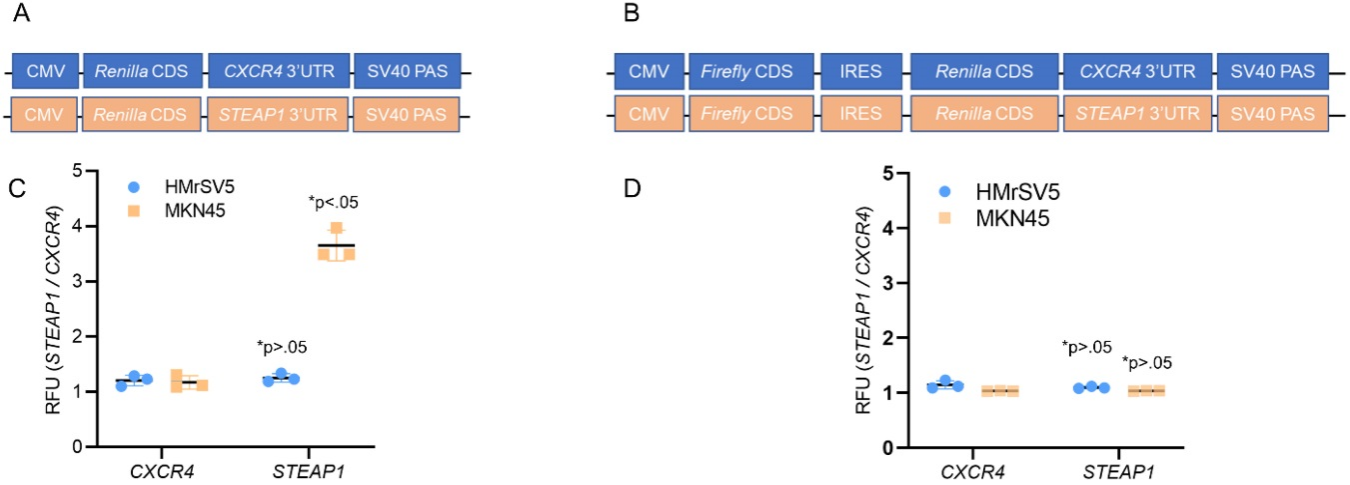
***STEAP1* expression is regulated at the translation initiation stage. (A)** Reporter assay quantifying the relative Renilla luciferase expression from *STEAP1* 3' UTR luciferase reporters in the tumor cell line MKN45 relative to HMrSV5 cells. **(B)** Reporter assay quantifying the relative Renilla luciferase expression from the indicated 3' UTR luciferase reporters driven from an internal ribosomal entry site in the tumor cell line MKN45 relative to HMrSV5 cells. In **A** and **B**, Firefly luciferase expression was used for normalization, and data were reported as folds over *CXCR4* reporter. *P* value shown in each case is for *STEAP1* reporter compared to *CXCR4* reporter in the same cells.

We next investigated the potential contributions of translational initiation and translational elongation to this increased translational expression of STEAP1. We fused the 3' UTRs of *STEAP1* mRNA downstream of a sequence that coded for *Renilla luciferase* in pRL-CMV CXCR4 6x reporter plasmid (Fig. 2A). We next built a construct in which the same CMV promoter was utilized to drive expression of bicistronic construct in which the firefly luciferase coding sequence was followed by the EMCV virus internal ribosome entry site (IRES), the *Renilla luciferase* ORF and *STEAP1*-containing 3' UTR (Fig. 2B).

Transfection of the *STEAP1* 3' UTR reporter into HMrSV5 cells (normal) and MKN45 cells (tumor) revealed a significant increase in reporter activity specific in the MKN45 cells (Fig. 2C). No difference in *CXCR4* 3'UTR reporter was observed between HMrSV5 and MKN45 cells (Fig. 2C). This increase was independent of any difference in relative mRNA expression of the reporters when the two cell lines were compared (*data not shown*). In contrast, comparison of Renilla luciferase expression derived from bicistronic *CXCR4* or *STEAP1* reporters showed no significant differences in Renilla reporter expression between the MKN45 and HMrSV5 cell lines (Fig. 2D). Again, relative levels of the mRNA encoded by each of these reporters were essentially unchanged in each cell line (*data not shown*). These results indicated that *STEAP1* mRNA is being regulated at the levels of 5' 7mG cap-dependent translational initiation.

**Figure 3:**
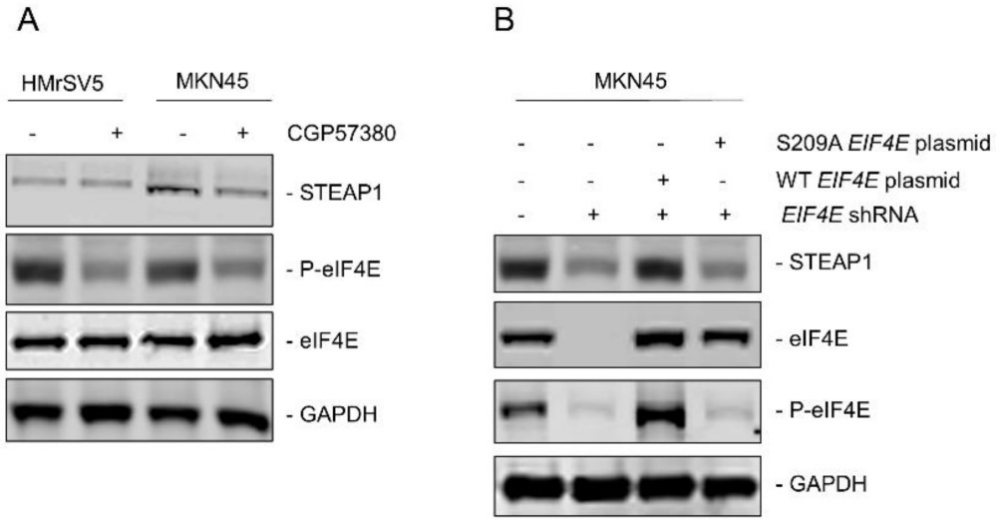
** Upregulation of STEAP1 expression is dependent on eIF4E and its ability to be phosphorylated. (A)** HMrSV5 and MKN45 cells were treated with MNK inhibitor CGP57380 for 24 hours. Lysates were probed with indicated antibodies to confirm inhibition of P-eIF4E and its effect on STEAP1 expression. **(B)** MKN45 cells were transduced with shRNA targeting the 3'UTR of *EIF4E*. Stable transductants were transfected with either wild-type or S209A mutant *STEAP1* expressing plasmid and stable clones generated. Lysates were immunoblotted with indicated antibodies to confirm eIF4E re-expression and its effect on steady state expression of STEAP1. In both **A** and **B**, blots were probed with for GAPDH as control All experiments were done at least three times and representative blots are depicted.

Since, it has been shown that phosphorylation of eukaryotic initiation factor 4E (eIF4E) at serine 209 can drive metastasis in different tumor models 25 and regulation of *STEAP1* was happening at the translational initiation stage, we next determined if eIF4E dependent mechanism was involved in peritoneal metastasis in gastric cancer patients. MNK is known to phosphorylate eIF4E at serine 209 residue 25. We first treated HMrSV5 and MKN45 cells with the MNK inhibitor CGP57380 (10 µM) for 24 hours. Lysates obtained from untreated and CGP57380-treated HMrSV5 and MKN45 cells were immunoblotted with anti-P-eIF4E antibody. MNK inhibitor robustly downregulated P-eIF4E in both cell types without affecting the expression of total eIF4E. Treatment of MKN45 cells with CGP57380 also resulted in significant downregulation in steady state expression of STEAP1 (Fig. 3A). This indicated that translation of STEAP1 might be regulated by eIF4E-dependent translation initiation mechanism.

To further confirm the requirement of phosphorylated eIF4E for translation of STEAP1, we made different variants of the MKN45 cells. First, endogenous eIF4E was knocked down using shRNA targeting the 3'UTR of *EIF4E* (MKN45-4E KD). Successful knockdown was verified by immunoblotting (Fig. 3B, second panel from top). Next, either wild type or S209A-mutant *EIF4E* was transfected into the MKN45-4E-KD cells (MKN45-4E-KD/WT 4E and MKN45-4E-KD/S209A 4E) and successful overexpression was verified by immunoblotting (Fig. 3B, second panel from top). P-eIF4E levels were also tested in these aforementioned MKN45 cell variants (Fig, 3B, second panel from bottom). Silencing of endogenous *EIF4E* significantly downregulated STEAP1 protein expression in the MKN45 cells (Fig. 3B, top panel-second lane from left). STEAP1 expression was rescued following overexpression of wild type *EIF4E* (Fig. 3B, top panel-third lane from left), but not following overexpression of the phospho-dead S209A mutant *EIF4E* (Fig. 3B, top-panel, right most lane). These results confirmed that P-eIF4E is regulating translation initiation of STEAP1 in the MKN45 cells.

**Figure 4:**
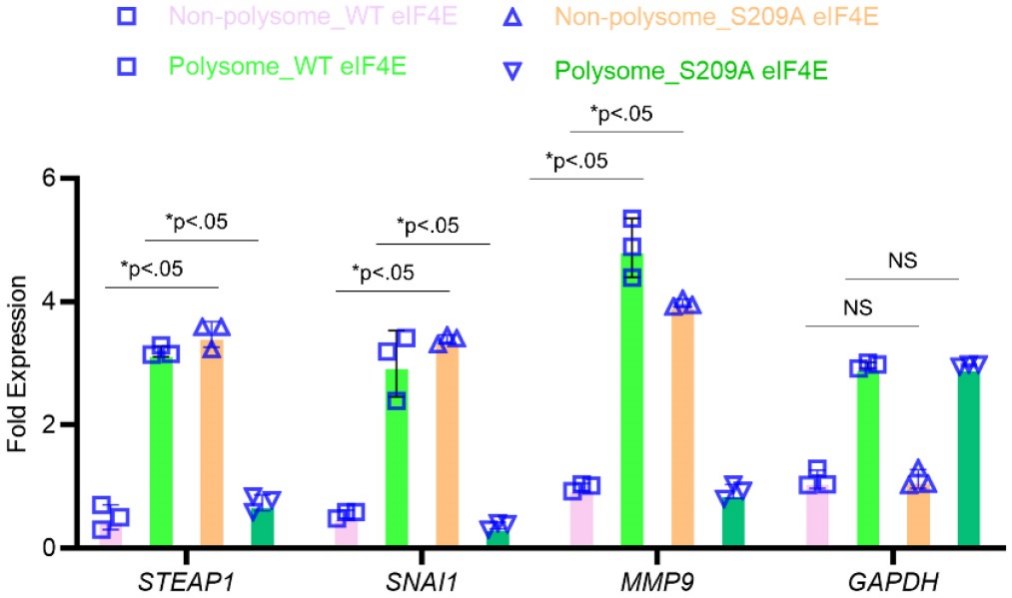
***STEAP1* expression is regulated at the level of translation by p-eIF4E.** The MKN45 cell variants overexpressing wild type or S209A mutant form of eIF4E were subjected to polysome profiling. qRT-PCR performed on RNA isolated from the resultant fractions were used as template to probe for relative ribosome enrichment of *STEAP1*, *SNAI1*, *MMP9*, and *GAPDH*. Data is presented as means ± standard deviation of 3 replicates (individual data points are depicted), each done in triplicate.

To confirm that P-eIF4E is mediating translational upregulation of *STEAP1*, we performed polyribosomal profiling on the MKN45 cell variants. Global translational was not affected in the MKN45 cells expressing wild-type or S209A eIF4E mutants (*data not shown*). Quantitative real-time PCR on polysomal fractions showed similar translation patterns of housekeeping gene *GAPDH* (Fig. 4). *SNAI1* and *MMP9*, known metastasis inducers and known to require P-eIF4E, was sequestered to the non-polysomal fractions in the S209A MKN45 cells (Fig. 4). Similarly, *STEAP1* mRNA was sequestered to the non-polysomal translationally inactive pool in the S209A MKN45 cells but was in polysome pools in the wild-type *EIF4E* expressing MKN45 cells (Fig. 4). Our overall results thus show that phosphorylated eIF4E potentiates *STEAP1* translation at the initiation step in peritoneal metastatic tissue.

## Discussion

Post-transcriptional regulation of gene expression in cancer progression is well documented 26, and have led to two regulatory roles for this process in cancer 27, 28. For one, in response to stress cancer cells act to ensure that only pro-survival proteins are readily translated to improve cancer cell survival. Secondly, processes that increase the number of translation initiating proteins ultimately lead to a loss of control over the cell cycle, allowing cancer cells to grow without constraints.

Through these two mechanisms, translational regulatory mechanisms mediate changes in cancer cell proliferation and survival - fundamental processes that directly correlate with the aggressiveness of disease. Given these precedents, it is reasonable to hypothesize that such translational programs also regulate the peritoneal metastasis of gastric cancer. And our results suggest that *STEAP1* is a central effector in this pathway.

Regulation taking place at the level of mRNA translation can occur via multiple mechanisms. For example, post-translational modification of the eukaryotic translation initiation factor 2 α-subunit (eIF2α) is generally regarded to have broad effects on the translational machinery 29. Conversely, regulation of more specific groups of transcripts may be mediated by shared *cis*-regulatory elements that may be contained in the in the 5' UTRs, 3' UTRs, or even protein coding sequences of these transcripts. In this latter case, regulation of these sets of transcripts is mediated by the binding of one or more distinct *trans* factors to these commonly shared cis-elements 30-36. It will be important to define the *cis* and *trans* factors for *STEAP1*'s translational regulation.

The data we present is most consistent with a model involving 5' 7mG cap-dependent translational initiation. Whether this increase in translational initiation of *STEAP1* are prevalent among a cohort of transcripts need to be determined. Our data shows that the potentiation of translation occurs due to enhanced binding of initiation factors such as eIF4E 25.

Work by other groups has shown that phosphorylation of eIF4E is required for EMT and metastasis via translational control of a subset of EMT inducers including *SNAI1* and *MMP*^25^. eIF4E is also able to regulate, in a dose-dependent fashion, a distinct network of mRNAs, functionally induced by oncogenic transformation, which share a common signature located within their respective 5' UTRs 35. These studies support the notion that both eIF4E expression levels and post-translational modification of this protein regulate translation initiation programs of specific networks of mRNAs encoding drivers of various stages of cancer development and progression. It might be possible that translational upregulation of *STEAP1* mRNA may directly impinge on or participate in these mechanisms, and this model will certainly be directly tested in future studies. It will be important to validate our findings of eIF4E-mediated translational upregulation of *STEAP1* in an independent cohort. One caveat is we can take advantage of publicly available dataset to perform the analysis since these datasets do not have protein expression data set. Furthermore, we did not see any significant correlation of STEAP1 expression with any clinicopathological characteristics. Part of this reason is perhaps due to the small number of patients included in the current study. We are currently obtaining more patients in our ongoing study and hopefully will be able to validate findings from the current study in a larger patient cohort which will also allow us to establish if there is any correlation of STEAP1 expression with clinicopathological features. However, the findings of the current study does argue in favor of pre-clinical endeavors of determining if inhibiting phosphorylation of eIF4E or its interaction with the translation initiation complex will be a viable therapeutic angle to treat peritoneal metastasis.
